# Recent breakthroughs in large-scale integrated photonic computing

**DOI:** 10.1093/nsr/nwag315

**Published:** 2026-05-27

**Authors:** Xinyu Liu, Shiyue Hua, Shanshan Yu, Huaiyu Meng, Yichen Shen

**Affiliations:** Xizhi Technology Co., Ltd, China; Xizhi Technology Co., Ltd, China; Xizhi Technology Co., Ltd, China; Xizhi Technology Co., Ltd, China; Xizhi Technology Co., Ltd, China

The rapid expansion of artificial intelligence (AI) into domains such as medical diagnosis, autonomous driving, and scientific discovery has driven a dramatic growth in computational demands [1,2]. Beyond standard AI workloads, emerging applications such as ultra-wideband microwave signal processing and real-time image processing in autonomous systems impose stringent ultra-low-latency requirements for handling massive data throughput [3]. Under these conditions, conventional electronic computing architectures are increasingly constrained by fundamental limits in performance scaling and energy efficiency [4].

Photonic computing has thus emerged as a promising paradigm to overcome these limitations, exploiting the intrinsic advantages of photons, including low latency, high bandwidth, massive parallelism, and superior energy efficiency [5]. To enable practical deployment, it is essential to construct photonic computing systems that integrate high computational power with robust programmability. As the scale expands, computing power and energy efficiency improve rapidly. Meanwhile, processing latency increases slowly and linearly with system size, in contrast to electronic architectures where multi-cycle operations lead to cumulative latency. This scaling behavior enables photonic processors to deliver orders-of-magnitude improvements in computational throughput and energy efficiency. Beyond intrinsic performance incentives, the escalating complexity of AI models and real-world workloads further motivates the pursuit of large-scale, highly integrated photonic computing systems as a viable and manufacturable solution.

Integrated photonic chips are crucial for commercializing laboratory prototypes (Fig. 1a). Currently, photonic computing exhibits distinct development paths in academia and industry. Academia explores diverse architectures, which can be broadly characterized by trade-offs among programmability, integration density, and energy efficiency [6,7]. Mach–Zehnder interferometer (MZI) arrays are distinguished by their ability to perform reconfigurable linear computations through optical interference, enabling highly programmable and universal transformations. Despite their relatively large physical footprint, which constrains integration density, these systems are particularly well suited for flexible and general-purpose AI workloads. In contrast, microring resonator (MRR) arrays exploit optical resonance to enable highly compact implementations and support wavelength-division multiplexing. Despite their susceptibility to thermal fluctuations, their high integration density makes them particularly well suited for compute-intensive applications. Diffractive optical networks perform computation through light diffraction and propagation, resulting in extremely low static energy consumption. Their limited reconfigurability, however, constrains their applicability, rendering them most effective for task-specific image processing. Finally, phase-change material (PCM)-based photonic devices rely on reversible structural transitions to enable non-volatile weight storage with minimal power consumption. Current challenges, including limited switching speed and endurance, restrict their performance. Nevertheless, these devices hold significant promise for energy-efficient edge inference applications [8,9].

**Figure 1.**
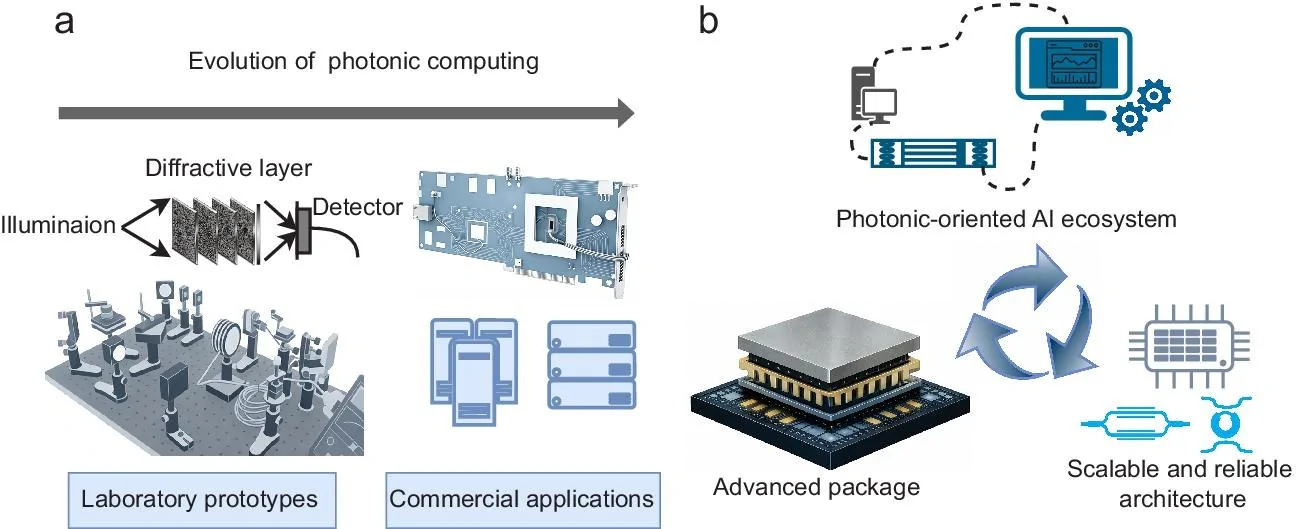
Evolutionary trajectory and scaling challenges of photonic computing. (a) Architectural transition from discrete-component laboratory prototypes to highly integrated, large-scale commercial systems. (b) Three key technological dimensions under active investigation for system scalability.

In contrast, industry has largely converged on an architecture centered around ‘large-scale integrated photonic-electronic hybrid,’ focusing on translating technological potential into manufacturable and highly reliable systems. A defining moment for this field arrived in early 2025 when leading industrial players, Lightelligence and Lightmatter, concurrently published milestone findings in *Nature*. This event collectively signaled that photonic computing technology has entered a critical transition from laboratory prototypes to industrial applications.

Lightelligence provided detailed technical specifications and system-level performance data for PACE (Photonic Arithmetic Computing Engine), a 64 × 64 optical matrix–vector accelerator comprising more than 16 000 photonic components co-integrated with a 28-nm complementary metal-oxide-semiconductor (CMOS) control chip through 2.5D hybrid packaging [10]. By flip-chip bonding the photonic and electronic integrated circuits, control signal sources are positioned mere

micrometers from photonic components, minimizing parasitic capacitance and facilitating exceptional system scalability. Furthermore, the adoption of an incoherent photonic computing architecture yields critical advantages, including simplified high-precision calibration and shorter coherent paths that significantly enhance temperature insensitivity. Consequently, large-scale matrix-vector multiplications can be executed on PACE with an ultralow computational latency of 3 ns per cycle. To maintain computational integrity and sustain a robust 7.61-bit average accuracy, PACE leverages numerical-to-digital-to-analog converter (DAC) lookup tables (LUTs) alongside open-loop thermoelectric cooler (TEC) thermal regulation. Notably, to facilitate solution search in heuristic algorithms, the system actively regulates dynamic noise through laser power and transimpedance amplifier (TIA) tuning. Ultimately, this combination of high precision, deliberate noise injection and ultralow latency empowers PACE to efficiently execute heuristic recurrent algorithms for Ising problems. As a result, PACE achieves reliable convergence with computation times two orders of magnitude faster than commercial GPUs.

While PACE emphasizes ultralow latency and physics-driven acceleration for optimization problems, Envise developed by Lightmatter demonstrates the feasibility of near-digital-precision, general-purpose AI inference [11].

The system integrates four 128 × 128 photonic tensor cores (PTCs) with 12-nm CMOS control logic through 3D-stacked electronic interconnection. At the algorithm level, Envise adopts an adaptive block floating-point (ABFP) numerical format tailored to the PTCs architecture, bridging the gap between high-precision requirements of AI models and the fixed-point constraints of analog converters. ABFP aligns well with the quantization bit depths of DACs and analog-to-digital converters (ADCs) by allowing data blocks to share a common exponent while leveraging adaptive scaling and hardware gain factors. To further mitigate accuracy degradation arising from analog noise in complex workloads, this approach is combined with quantization-aware training (QAT), enabling models to adapt to low-precision effects and maintain robust inference performance. Through such hardware-algorithm co-design, Envise executes AI models, including ResNet, BERT, and reinforcement learning tasks, with near-digital precision. Operating at 65.5 TOPS while consuming only 1.6 W of optical and 78 W of electrical power, it impressively achieves 97.8% of FP32 accuracy on CIFAR-10 using ResNet-18 without retraining.

Collectively, these systems establish both the functional versatility and superior performance potential of large-scale photonic computing beyond conventional electronic accelerators. The evolution from the early-stage prototypes to PACE and Envise marks a critical inflection point, signifying the emergence of system-level photonic processors capable of addressing both application-specific optimization problems and general-purpose AI workloads. Moreover, these systems have independently explored and validated viable technical pathways for high-density optoelectronic hybrid packaging, laying a practical engineering foundation for subsequent generations and scalable advancement. The introduction of more advanced packaging technologies, such as 3D through-silicon vias (TSV) and hybrid bonding, is expected to further enhance the integration density of large-scale integrated photonic computing systems, thereby unlocking greater potential for photonic computing.

Despite significant advances in hardware development, several issues remain worthy of discussion (Fig. 1b):

- Direction of technical evolution. While current industrial systems demonstrate the feasibility of large-scale photonic accelerators, the long-term technological roadmap continues to evolve with diverse development trajectories. Regarding material platforms, silicon photonics offers a stable and mature commercial foundation, whereas emerging platforms including thin-film lithium niobate (TFLN), barium titanate (BTO), and magneto-optic materials exhibit significant potential in terms of modulation efficiency, optical loss, and non-volatile functionality [12–14]. Architecturally, as integration density continues to increase, navigating the trade-offs among precision, scalability, power efficiency, and reconfigurability will strongly dictate future design choices.
- Ecological and algorithmic barriers. Photonic computing inherently relies on noise-sensitive analog computation, whereas the current AI ecosystem is deeply optimized for digital hardware, supported by mature software stacks, numerical formats, and training paradigms. This mismatch necessitates a fundamental rethinking of the algorithm–hardware interface. On the algorithm side, specialized numerical formats, such as ABFP, together with noise-aware training, can mitigate hardware-introduced errors and enable accurate model execution under limited precision. Furthermore, heterogeneous photonic-electronic integration offers a promising pathway to bridge this divide. Photonic cores can efficiently perform compute bound tasks such as general matrix multiplication (GEMM), while electronic processors handle control intensive, nonlinear tasks.
- Industrialization challenges. The scaling of photonic computing hardware is influenced not only by intrinsic physical factors, such as optical loss and noise, but also by industrialization requirements, including advances in yield, packaging, and precision control strategies for long-term computational integrity. Addressing these challenges is essential to enable the deployment of complex models and to ensure the commercial competitiveness of photonic accelerators. Promising directions for future development include coordinated advances in photonic fabrication, heterogeneous integration, and system-level validation methodologies.

As an emerging computational paradigm, photonic computing offers transformative capabilities for high-throughput, energy-efficient, and ultralow-latency acceleration beyond the limits of conventional electronic architectures. Moving beyond proof-of-concept demonstrations, the field is now entering a stage where system-level integration and cross-layer co-design will determine its practical impact. In this context, the challenges associated with scaling should be viewed not as obstacles, but as critical opportunities for innovation. By jointly advancing algorithms, packaging technologies, and architectural design, future efforts can help bridge the gap between laboratory prototypes and real-world deployment. Such progress will not only enable scalable photonic computing systems but also define their foundational role in shaping the future landscape of next-generation computing infrastructure.
